# Spatial and Temporal Complexities of Reproductive Behavior and Sex Ratios: A Case from Parasitic Insects

**DOI:** 10.1371/journal.pone.0019438

**Published:** 2011-05-10

**Authors:** Katharina Dittmar, Solon Morse, Matthew Gruwell, Jason Mayberry, Emily DiBlasi

**Affiliations:** 1 State University of New York at Buffalo, Department of Biological Sciences and Graduate Program of Ecology, Evolution and Behavior, Buffalo, New York, United States of America; 2 Penn State Erie, The Behrend College, Erie, Pennsylvania, United States of America; University of Plymouth, United Kingdom

## Abstract

**Background:**

Sex ratios are important empirical data in predicting sex allocation strategy and selection in populations. Therefore, they should be sampled at crucial developmental steps before and after parental investment. In parasites with free-living (off-host) developmental stages the timing and method of sampling is not trivial, because ecological niches are frequently poorly known. Consequently, information is scarce for sex ratios of these parasites between conception and sexual maturity. Often, only data from adult parasites are available, which usually were collected from the parasite's hosts. Generally, these ratios are assumed to represent operational sex ratios.

**Methodology/Principal Findings:**

We here report three years of empirical data on population sex differentials from a bat ectoparasite (*Trichobius frequens*) with off-host developmental stages. At emergence these parasites exhibit a significant and seasonally stable female biased sex ratio. This bias is lost in the adult population on the roosting host, which shows sex ratios at equality. This is best explained by a behaviorally driven, sex-dependent mortality differential. Because consistently only subsets of females are available to mate, the operational sex ratio in the population is likely male biased. Host capture experiments throughout the day show a statistically significant, but temporary male excess in bat flies on foraging bats. This phenomenon is partly driven by the diurnal rhythms of female larviposition, and partly due to parasites remaining in the bat roost during foraging. Because most previous research in bat flies is based only on foraging bats, female contributions to physical sex ratios have been underestimated.

**Conclusion/Significance:**

Our results highlight the importance of detailed natural history observations, and emphasize that ignoring the spatial and temporal heterogeneity of reproduction in any organism will lead to significant empirical sampling errors of sex ratios, and may obscure operational sex ratios.

## Introduction

Most dioecious species ultimately produce near equal, or equal numbers of males and females in a population (evolutionary stable strategy, ESS). This general principle was observed by Darwin, and later explained and theoretically refined by Düsing, Fisher and Hamilton (among others) [1]–[3]. In natural populations it is likely that biased sex ratios are observed, as the conditions required for complete equality are rare. In fact, observations of skewed sex ratios may be a first, albeit purely descriptive hallmark for conditions or pressures warranting differential parental investment.

Because of relative ease of assessment, the majority of sex-ratio observations in natural populations (including parasites) are made from tertiary ratios, which describe the population sex differential of reproductive adults. Tertiary ratios are physical ratios, and since parasite reproduction is understood to be spatially tied to the host, they are often assumed to also reflect operational sex ratios (OSR). Operational ratios are defined as the ratio of actively reproducing males to fertilizable females, and thus may only encompass a subset of all adults in the population (i.e. effective population size) [4].

Sex-ratios of adults are but one data point in understanding the dynamics of resource allocation to either sex. As such, primary and secondary ratios are also important, because they more directly reflect sex determination outcomes, and female resource investment. The primary ratio is understood as the male/female ratio at conception; secondary ratio refers to that ratio at birth. Historically, these terms and definitions were coined in the context of vertebrate development, which may not have obvious analogs in organisms with different life histories. Since the parasite studied here is a viviparous, holometabolous insect, we refrain from using traditional terms, and rather explain each sampling point in the context of the reproductive biology of our system.

In insects, research on sex ratios and sex allocation theory is most prolific for social systems, and parasitoids [5], [6]. For ectoparasitic insects (our system) comparatively little empirical and theoretical research is available [7]–[10]. Some ectoparasites (e.g., lice, fleas, ked flies) have been reported to show a predominately female physical bias in sex-ratios of the adult population [11]–[13]. These observations have lead to a generalization regarding a correlation between ectoparasitism and female sex bias trends [12], [13]. Potential explanations for this pattern are female longevity, active, dispersal prone males, or higher male mortality due to differential grooming. Very little information is available for pre-adult ectoparasite sex ratios. This problem is especially apparent for ectoparasites with free-living (off-host) developmental stages, because their ecological niches are unknown, or difficult to access for sampling.

Based on the scarcity of details, further research on ectoparasitic insects seems to be warranted. We here choose *Trichobius frequens* (Streblidae) as our study system - a Neotropical bat fly with off-host developmental stages. Bat flies are Diptera closely related to ked flies (Hippoboscidae), and tse-tse flies (Glossinidae) [14]–[16]. All bat flies are obligate blood-feeders, exclusively adapted to bats. Like most ectoparasites they exhibit a suite of characteristic phenotypes, such as wing, and eye reduction, as well as specialized hold-fast structures [17], [18]. Males and females of *T. frequens* possess wings, and are capable of flight and local dispersal.

Female bat flies are viviparous (live-bearing), producing a single offspring each reproductive cycle [8], [17], [18]. Thus, their reproductive rate is low. Larvae grow and molt within the uterus, and are nourished by modified female accessory glands. The interval of consecutive larvae per female is unknown for *Trichobius*. Other bat flies have been observed depositing larvae every 3 to 9 days [19]. Larvae are deposited in a late stage (prepupa) on a substrate away from the host [8], [20].

Larviposition requires females to actively leave their hosts, and find a suitable habitat for offspring development. Because *T. frequens* parasitizes cave roosting bats, the pupal habitat is the cave wall. Pupae of *Trichobius* have been observed at considerable distance from the actual bat roosts, including cave entrance areas [8]. No offspring are deposited outside the cave, which is consistent with general bat fly ecology. Occasionally, as is the case in *T. frequens*, bat fly pupae are deposited in conspicuous clusters on the cave wall (pupal deposition field) [20]. At this time it is unclear whether this is a species-specific trait, and/or related to local roost ecology.

Duration of pupal development varies with species, as well as environmental conditions such as temperature and humidity. Some bat flies can enter diapause within their puparium, but it is unknown whether this also applies to *T. frequens*. Upon emergence, and before their first blood meal, adult bat flies are referred to as *tenerals*.

After larviposition, the female returns to a host bat. Females may have multiple offspring over the course of their lives (iteroparous), and thus have to leave the host repeatedly. Males mostly stay on the host, or in the immediate vicinity of a roosting bat. Only anecdotal records have reported male bat flies at considerable distance off the host [8], and they have not been observed on the pupal fields [20].

Copulation of *Trichobius* has been reported on roosting bats during the day, or occasionally in the roost at night [8], [21]. Once fertilization occurs, females are not fertilizable until after larviposition [8], [21], although males have been observed trying to mate females carrying larvae [19]. It is assumed that a male can inseminate multiple females, preferring virgin females (tenerals) as mates [8]. Tenerals presumably reach sexual maturity after their first blood meal, thus no mating occurs on the pupal field directly after emergence, but rather on the host in the roost [19], [21]. Female bat flies posses a spermatheca [8], yet it is unclear to what extent they are capable of sperm storage.

Only subsets of parasites reproduce and emerge every day, and overlapping parasite populations exist in bat roosts [7], [8], [22]. Deposition of offspring and pupal emergence show a distinct diurnal pattern in all bat fly populations. Depositing females are known to leave the host at the beginning of the bats' foraging activity. Tenerals have been observed to emerge during the time of the hosts' return [8], [19], [21]. However, species-specific dynamics of this behavior are largely unstudied.

Specific data on bat fly sex-ratios are rare, and at times confounding. No data exist on sex ratios at conception. Only one publication, using two small samples, speaks to their ratios at emergence, observing no aberrance from unity [23]. Other research reporting on reproductive parasite ratios from collections off foraging hosts finds evidence for equality, female, or male bias, depending on species [7], [13], [24]. Marshall [13] reports equal ratios for most bat fly species in his data set, female biased adult ratios for nycteribiid bat flies, and a male bias for most Neotropical bat flies. The latter findings are also supported by Dick and Patterson [7], reporting a significantly male-skewed, seasonally independent sex ratio on a third of bat fly metapopulations from the to date largest available collecting effort [Smithsonian Venezuela Project] [25]. Differential host grooming of larger females was offered as an explanation for the observed male skew [7].

Given their complex developmental cycles, and taking into account missing data and contradictory evidence presented in previous studies, it is our goal to extend sex ratio analysis in parasites, using bat flies as a specific example: 1) We test if the sex ratio at emergence differs from the ratio in the reproductive population on the hosts. Comparing these ratios will offer some insight into selective pressure from conception to deposition, as well as the magnitude and skew of mortality after parental investment. 2) We explore the influence of the larger environment on parasite mortality. Commonly, only host-parasite interactions are cited for parasite mortality (i.e., host grooming). However, given the knowledge that females as well as tenerals find themselves away from the roost and the host, it is likely that processes unrelated to the host influence mortality. Because females are iteroparous, and repeatedly leave the host, we expect them to be more affected by these influences than are male flies. 3) We test if the observable adult sex ratio on the host is influenced by temporal and spatial aspects of reproductive behavior. In other words, in the absence of influence of parasite reproductive behavior, we would expect equal sex differentials on foraging and roosting bats from the same cave on the same day. Likewise, if there is an influence one could expect that only sub-populations of parasites are on hosts, which in fact do not represent true population sex ratios. Embedded within the framework of the first three questions, we also explore the assumption that physical parasite sex ratios observed on the host should be a proxy for operational sex ratios.

## Methods

### 1. Location, hosts, and parasites

**Figure 1:**
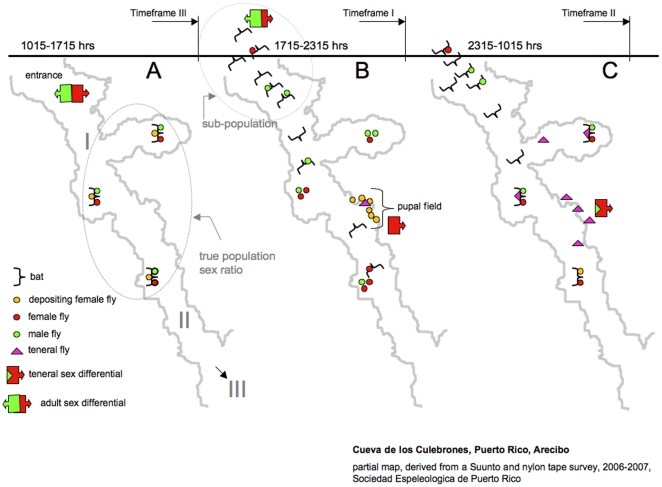
Schematic overview of bat fly location during 24 hrs. Location of adult bat flies, teneral flies, and bat hosts during the three sampling timeframes (1,2,3) throughout a day is shown (A,B,C). (**A**) During Timeframe III almost no host activity can be detected, and an equal sex ratio is observed on roosting hosts, representing the physical true population ratio. (**B**) During Timeframe II bat hosts are exiting the roost, and male skewed parasite ratios can be observed on exiting foraging bats (subpopulation). Female depositing flies can be observed on the pupal field. No males are observed in this area. Some males and females are observed in the roost, without bat hosts. (**C**) Timeframe I, is marked by the emergence of tenerals from the pupae, which show a female biased sex differential. The cave outline was extracted from the current Culebrones cave map (Sociedad Espeleologica de Puerto Rico, Suunto and nylon tape survey, 2007), and only shows the first two rooms of the cave. The back area (III) is roost to five other species of bats known from this cave.

**Figure 2:**
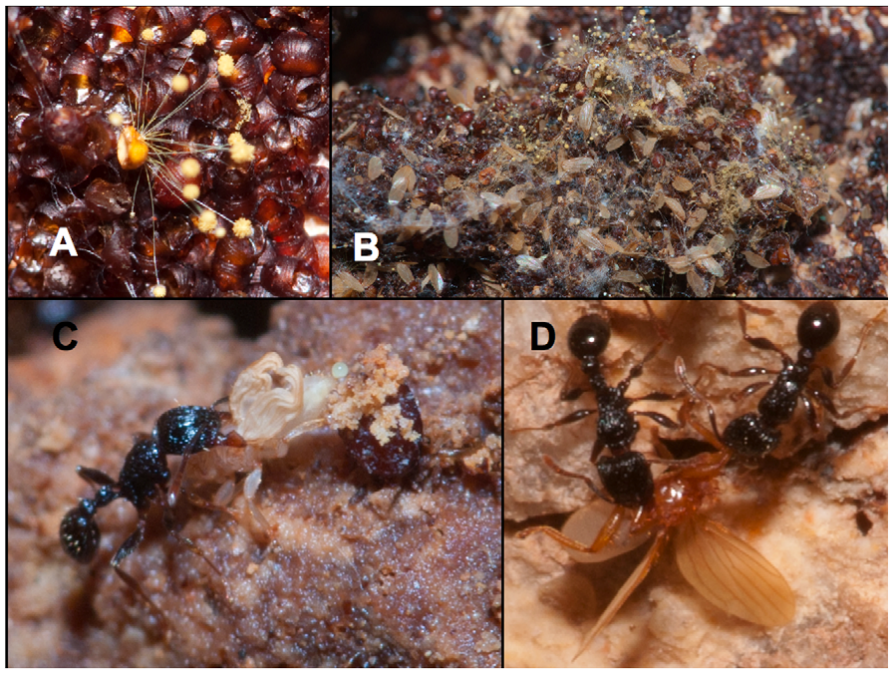
Predation on pupal field. (**A**) Pupa on the deposition field attacked by a fungus. (**B**) Debris of bat fly wings (from predation), and fungi in front of a nest entrance of *Tetramorium* sp. (**C**) *Tetramorium* worker ant capturing a freshly emerged teneral *Trichobius frequens* (see unfurled wing tips). (**D**) *Tetramorium* worker ant capturing a female *T. frequens* after larviposition.

All studies were carried out in Cueva de los Culebrones, Mata de Platano Field Station in Puerto Rico (18.44°N, 66.70°W) between 2008 and 2010 [06/19–06/26/08; 10/27 to 10/31/09; 02/07–02/13/10; 05/15–05/23/10]. The cave starts out with two rooms (entrance room I and II), accessible by a small, steep chute (Figure 1A). From room II, the back area (III) can only be entered or exited through a relatively narrow opening. The back room eventually dead ends, and harbors the majority of the estimated 300.000 bats occupying this cave. Six species are represented, namely: *Pteronotus quadridens*, *P. parnelli*, *Erophylla sezekorni* ( = *bombifrons*), *Brachyphylla cavernarum*, *Monophyllus redmani*, and *Mormoops blainvillii*. Temperature and humidity fluctuations were recorded using LogTag (Haxo-8) technology over an entire year (2009–2010). In one of the entrance rooms a pupal deposition field can be observed ([20]; Figure 2A, B), which consists mainly of pupae of the bat fly *T. frequens* Peterson & Hůrka, 1974. *T. frequens* is frequently confused with *T. truncatus*, which has also been reported from Puerto Rico. However, the small size, the distinct shape of the sternopleuron, as well as the pattern and size of the scutellar bristles clearly indicate *T. frequens*, thus representing a new record for Puerto Rico [26]. Occasionally, pupae of *Nycterophilia* sp. get deposited, but are easily distinguishable by shape and size.

The dimensions of the pupal deposition field were measured with a Ryobi Tek4 Digital Multimeter (RP4010). To estimate the number of pupae on the field, several one square centimeter plots were marked and completely cleared of pupae. Pupae were collected and counted. The average number of pupae per cm^2^ was computed and counted against the entire area of the field. In Culebrones cave their main hosts are *E. sezekorni*, which roost in entrance rooms I and II in the cave (Figure 1 A–C). Based on observations in the cave (bell hole counts) and harp trap captures we estimate the number of individuals at ca. 70.000, which is down from populations before hurricane George in 1998. *T. frequens* has also been reported from *Brachyphylla* sp., which also occur in this cave [26]. However, their roost is very small, and in the back of the cave. Based on our observations, their parasite populations deposit locally, in the roost, and don't use the major pupal field in the cave entrance area. Therefore we assume that they have no numerical contribution to the main *T. frequens* population on *E. sezekorni*.

### 2. Ratios at Emergence

To assess sex ratios at emergence, before tenerals set off to find a host, bat fly pupae were sampled at random from the pupal deposition field. Thus the sampling represents pupae of different ages. Viability of pupae was assessed by visual inspection. All pupae were subsequently reared out under controlled conditions in an insect rearing chamber (Percival Scientific, 66 Series) at SUNY Buffalo. Rearing conditions (temperature and humidity) were optimized based on LogTag measurements from the cave (see point 1, above). Every day eclosed teneral flies were collected and pupal mortality rate was recorded. Tenerals were killed with ethyl-ether, then deposited in 96% ethanol, and slide mounted using PVA medium (BioQuip). Slides were dried in a slide-oven, and flies were counted and sexed using an Omano OM334 trinocular stereomicroscope. Additionally, teneral sex ratios were recorded directly in their natural habitat from glue trap capture experiments (see point 3, below).

### 3. Assessing reproductive bat fly behavior/biology

**Figure 3:**
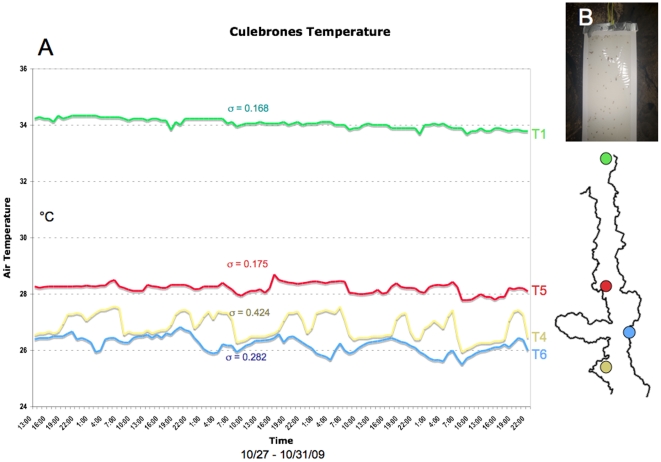
Methods of data capture. (**A**) Excerpt of temperature loggers (LogTag, Haxo 8), for 4 measuring points throughout the cave. T1 represents measurements in the main roost (room III); T4, and 6, are placed in the entrance area of room I; T5 represents the measurements on the pupal field (as per cave outline on the right). Graph was modified from Dittmar and Mayberry [46]. Standard deviations are shown for each logger. (**B**) Overview of a glue board, placed on the pupal field. Dark spots are captured bat flies.

Numbers of females away from the host per reproductive cycle were assessed by intercepting flies at the pupal field throughout a 24 hour cycle. Three time frames were chosen per day: Timeframe 1 - from 1715 hrs until 2315 hrs, timeframe 2 - from 2315 hrs until 1015 hrs, and timeframe 3 - from 1015 hrs until 1715 hrs. The choice of these intervals was guided by previous publications that recorded differential bat (host) and female bat fly activity throughout the day [8], [23], [27], [28]. Capture was accomplished using non-poisonous glue boards (Real Kill®, household glueboard; Figure 3B). Traps were tested *a priori* to ensure that they don't differentially attract bat flies, thus potentially biasing results. For the time series, traps were always placed in the same position, at two heights (1,65 m & 1,30 m) from the floor. Traps were placed facing the pupal wall to capture teneral (emerging) flies, and facing the cave to capture females arriving to larviposit. Captured females then deposited their pupa on the glue trap, which gave additional evidence for the presence of a female on a glue trap. All flies were counted per trap, and categorized by sex and developmental stage (teneral/adult). Teneral flies are easily distinguishable from other adults by their lighter color, and occasionally unfurled wings (Figure 2 C, D). Other glue boards were placed randomly throughout the cave, and in the vicinity of bat roost, to control for potential bat fly activity in the roost, away from the pupal field. To calculate the average number of depositing females (df_av_) on the pupal field per night, an average of all pupae per glue board was computed from 26 randomly placed glue boards from all 4 sampling periods (df_trap_). The dimensions of a trap (185 cm^2^) were measured, and counts per trap were then related to the entire pupal field (12.8 m^2^, df_av_ = df_trap_×691.89). We computed the average percentage of flies depositing per night, using the average numbers of female flies per roosting bat, which were extrapolated from the differential capture experiments. This number was multiplied by the population size of potential host bats. Bat fly behavior and predation events were observed and video-recorded at the pupal deposition field throughout the day (Figure 2 A–D).

### 4. Differential bat capture

To further explore the influence of a potential sample bias on sex ratios, host bats were captured at different times and cave locations throughout one day, in concordance with previously recorded diurnal bat fly and bat activity patterns (Figure 1 A–C). Specifically, bats were caught with a stationary harp-trap when leaving the roost to forage (1715 hrs–2315 hrs), as well as hand-netted inside the roost during the day (1100 hrs–1500 hrs, within timeframe 3). To ensure exhaustive sampling of bat flies, bats were immediately sacrificed at the capture site in the hand-net (permit DRNA2010-IC-030). This approach circumvents all handling of the bat, and prevents bat flies from escapeing. All ectoparasites were retained in 96% EtOH, and processed following the same procedures as outlined in section two. Female bat flies that were carrying offspring were identified based on their large and distended abdomen [8], [23]. Numbers of females with, and without extended abdomen were recorded.

### 5. Statistics

Samples per month, and foraging versus roosting bats were treated as independent. The significance of male versus female numbers per sampled population was tested with a chi-square test against an expected distribution of equality. Contingency table analyses were used to assess the significance of ratio differences per independent sample. Sex ratio biases within sampled populations were evaluated using nonparametric Wilcoxon tests. Correlations of male and female flies per host were tested using non-parametric Spearman rank tests. Analyses were carried out using the interactive SOCR (Statistics Online Computational Resource) tool of UCLA (http://www.socr.ucla.edu/SOCR.html), as well as SPSS Inc. (IBM, PASW Statistics 18.0). All statistical tests are two-tailed.

## Results

### 1. Basic environmental parameters

Cueva Culebrones is one of the hottest caves in Puerto Rico, with a maximum temperature at 34.5°C. Average temperature at the pupal deposition field is 28.3°C (σ = 0.175) (Figure 3 A). The size of the core pupal field is approximately 12.8 m^2^. The total number of pupae (eclosed and uneclosed) on the field is estimated at 6,934,980 (minimum), pointing to a continued use over multiple years.

### 2. Sex ratios at emergence

**Figure 4:**
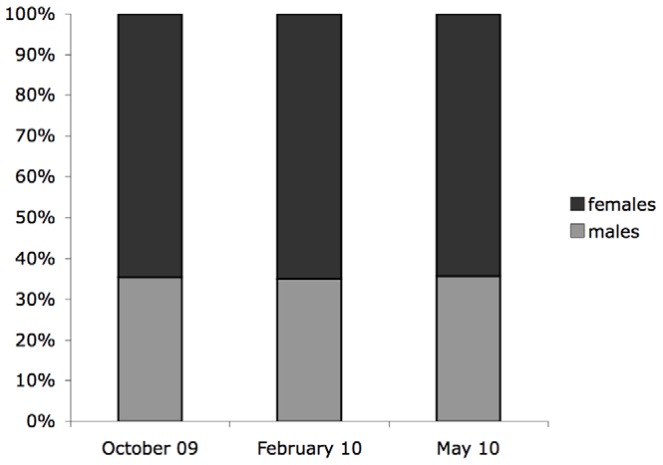
Sex ratio at emergence. Block diagram of percent male to female *T. frequens*, scaled to 100%, for three sampling months. All months show an excess of females (dark grey), and proportions are very similar throughout.

A total of 362 bat fly pupae of *T. frequens* were harvested from the cave's extensive pupal deposition field in October 2009, February 2010, and May 2010. Of 362 pupae reared out under controlled conditions, 56 did not eclose (15.5% mortality) (Table 1). Further observation of these pupae showed that they either stopped developing (reason unknown), or were attacked by a commonly observed fungus (Figure 2 A,B). Since all of these pupae originate from the pupal field, it is likely that the fungus was already present on the pupae upon collection. The mortality rate was relatively consistent in all three sample-batches (Table 1). Of the remaining 306 pupae, 198 eclosed as female tenerals, and 108 as males, effectively representing a sex ratio of 1∶1.83 towards females. This is significantly different from equality (χ^2^ = 26.471; p<0.001). Parsed out over three samples similar significance levels, and male/female ratios were observed for each batch, independent of sampling month (Table 1, Figure 4).

**Table 1:** Numerical results.

	October 09	February 10	May 10		February 10
A	Lab rearing			total	pupal field
male (N)	30	37	41	108	43
female (N)	55	69	74	198	76
total (N)	85	106	115	306	119
**sex ratio m∶f**	**1∶1.83**	**1∶1.87**	**1∶1.8**	**1∶1.83**	**1∶1.77**
chi-square	7.353	9.660	9.470	26.471	9.151
p-value	= **0.0069**	= **0.0019**	= **0.0021**	<**0.001**	= **0.0025**
	**dead pupae**				
total number	15	20	21	56	unknown
mortality %	15	15.87	15.44	15.5	unknown

B					
Wilcoxon	N of males	N of females	N total	Z	p
foraging bats	39, s = .58	24, s = .17	63	2.62	**0.0044**
roosting bats	25, s = 1.02	29, s = .65	54	0.89	0.1867
**OSR**				ratio m∶f	
roosting bats	25	12	37	**2.1∶1**	**0.033**

(**A**) Results from the pupal rearing experiments from lab reared specimens, collected in October 09, February 10, and May 10, as well as teneral count from the pupal field. Bold numbers identify sex-ratios, and significant p-values for the chi-square tests. (**B**) Results of the Wilcoxon rank sum test for samples from foraging, and roosting bats, as well as the chi-square test for the operational sex ratio (OSR). Bold numbers identify significant p-values.

From the glue boards 119 teneral flies were recovered (February 2010). Of these, 76 were identified as females, giving a ratio of 1.77 females for every male (Table 1). Again, this deviates significantly from an equal ratio (χ^2^ = 9.151; p = 0.0025). Furthermore, there is no significant difference between the ratio at emergence assessed from data the lab, versus the natural environment.

### 3. Reproductive bat fly behavior

**Figure 5:**
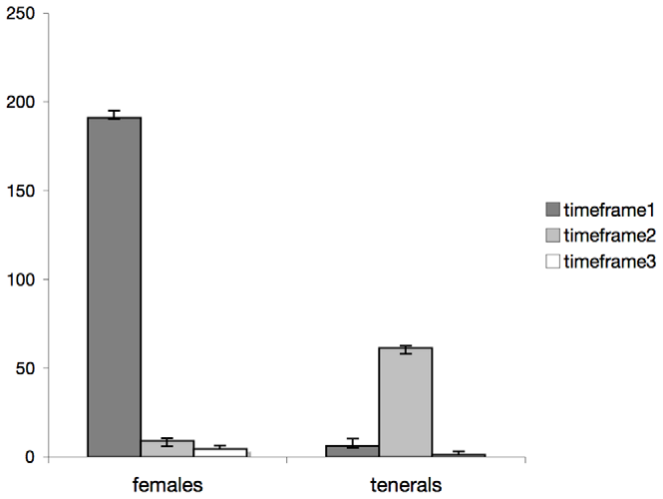
Fluctuations of adult females and tenerals throughout 24 hrs. Block diagram of the average numbers of depositing females, and tenerals of *T. frequens* from glue board time series on the pupal field, with standard error bars (95% confidence interval). Timeframe 1 (1715 hrs until 2315 hrs) shows high activity of depositing females, timeframe 2 (2315 hrs until 1015 hrs) represents the peak of teneral emergence, while almost no activity is observed during timeframe 3 (1015 hrs until 1715 hrs) for both tenerals, and depositing females.

The majority of bat flies (72.5%) were captured on the glue boards during timeframe 1 (1715–2315 hrs), coinciding with the foraging flight of bats (Figure 5). The age structure of the parasites represent on average 97% females with pupae, and 3% tenerals. Per glueboard, an average of 15 flies was estimated (df_trap_). The estimated average number of females on the entire pupal field during timeframe 1 per night (df_av_) is 10,378 (refer to Materials and Methods, point 3 for calculation). Given an expected average number of 2.6 females/bat in the roost (see point 4, below), 5.7% (for 70,000 bats) of all females are estimated to be relocated to the pupal deposition field during foraging activity. Observing the distribution of flies on each glue trap make it clear that flies were intercepted while flying, and did not walk to the pupal field (Figure 3B). During timeframe 2 glue boards yielded 25.7% (average) of all flies collected in a 24 hr cycle (Figure 5). Of these, only 15.8% represent females with pupae, and the overwhelming rest are teneral flies. Timeframe 3 yielded the lowest number of flies, with an average of 1.8% of the total, indicating almost no bat fly activity on the pupal field during daylight hours (Figure 5). All flies caught in this time were females with pupae, and likely represent early females seeking out the pupal field shortly before 1715 hrs. All glue boards that were placed throughout the cave in locations other than the pupal field captured no bat flies in any of the three timeframes. Therefore it is safe to assume that bat fly activity in the cave environment is restricted to the pupal field.

Visual inspection of the pupal deposition field during each of the three timeframes revealed three main sources of predation of bat flies, affecting adult female *Trichobius*, freshly deposited pupae, as well as teneral flies (Figure 2 A–D). These are: *Tetramorium* sp. (pavement ants, Myrmicinae, Formicidae), laelapid mites (*Stratiolaelaps* sp., Laelapidae, Gamasina), and entomopathogenic fungi. Predation of adult flies on the pupal field (females and tenerals) occurs only by ants, on a daily basis during timeframes 1 and 2 (Figure 2 C, D). Within a 30 minute observation window during timeframe 1, seven predation events of depositing females were observed. Ants either carried the captured flies into their nest, or devoured them in place. Ant predation is also evidenced by numerous bat fly wings discarded from the ants nest (Figure 2 B). Pupal predation is observed to mainly occur by stratiolaelapid mites, and fungi. *Stratiolaelaps* is known to be predatorial, facilitated by the presence of robust, snapping chelicerae, and bayonet-like corniculi [29]. Occasionally, freshly deposited bat fly pupae are carried of by ants into their nests.

### 4. Differential bat capture

#### Roosting bats

In the main *Erophylla* roost, eleven (11) bats were captured during daylight hours using sweep nets with extendable poles [June 2008 (3 adult males, 3 females post partum); May 2010 (3 adult males, 2 females post partum)]. Bats were captured from different spots, to randomize the sample, as *Erophylla* has a tendency to lek [30]. Compared to the total *Erophylla* population this is a small sample. The general inaccessibility of the roosts, and the bats escape reaction to roost disturbance make sampling extremely difficult, and did not allow for a more extensive sampling at this point. A total of 54 *T. frequens* were collected from these bats, with 29 being female flies, and 25 being male flies (4.9 flies/bat) (Table 1). Of the 29 female flies, 17 were identified with significantly extended abdomen, representing a mixture of flies with larvae at varying stages of development. Although there is a slight skew towards females in the physical ratio, this deviation is not significant (Wilcoxon Test, z = 0.89; p = 0.1867). Taking the “pregnant” females out of the equation to emulate the operational sex ratio, we find a clear, and statistically significant skew towards males, with a ratio of 2.1∶1 (p = 0.033) (Table 1). No significant abundance correlations between males and females could be detected (r_s_ = 0.1804, p = 0.297).

#### Foraging bats

Twenty *Erophylla* were sampled with a stationary harp trap intercepting bats exiting to forage (June 2008 (12); October 2009 (8)]. From these bats 63 flies (3.15 flies/bat) were recovered, representing 24 females, and 37 males, a bias which is significantly skewed towards males (Wilcoxon Test, z = 2.62; p = 0.0044). No influence of sampling size could be detected. The abundance of males was negatively, and significantly associated with the abundance of females (r_s_ = −0.314; p = 0.0089). None of the females captured from foraging bats were ready to larviposit, but all of them were with a developing offspring. The number of hosts with males outnumbering females did not differ from random (p = 0.197). However, average numbers of males and females per foraging bat were lower than those of roosting bats. Taking the mean number of males and females from roosting bats as a baseline, an average 55% of females, and 14% of males is estimated to be absent from foraging bats (35.7% total, estimation includes depositing flies).

## Discussion

From this study, we can derive trends that have implications for our general and specific understanding of parasite sex ratios dynamics.

### Dynamics unique to bat flies

#### Sex Ratio at Emergence

Since bat flies are holometabolous, and viviparous insects, it is useful to first define the crucial points of sex ratio sampling in terms of developmental stages. The sex determination system of bat flies is unknown, and thus, ratios at conception cannot be predicted. The ratio at larviposition is equally unknown, and will remain difficult to assess. The ratio at emergence from the pupal case has been sampled in this study, and it is clearly female biased. In the case of no sex-dependent mortality between larviposition and emergence, this ratio may serve as a proxy for the rate at larviposition (birth), but further research is necessary to explore this notion.

Biased sex ratios are the results of differential selective pressures on either sex, at different developmental stages [6]. A sex bias among later developmental stages in any holometabolous insect population may be explained by an already skewed ratio at conception, or an adjusted sex ratio immediately after conception. The most powerful causative agents for the latter are sex ratio distorters [6]. Previous publications, as well as ongoing research in our lab show the presence, and horizontal inheritance of *Arsenophonus* spp. in bat flies, as well as its immediate relatives, the Hippoboscidae [31], [32]. These bacterial endosymbionts are known to be male killers, and may play a role in producing a female bias at emergence [33]. Male killing endosymbionts often instigate selective abortion immediately after fertilization (early male killers). Thus, costs in the form of maternal resource loss and delay in reproduction are curbed [6], [34]. It is unclear whether *Arsenophonus* infection has a male killing effect in bat flies, and the selective drivers for a stable maintenance of this symbiont in the bat fly system are still being explored.

Another explanation for a female bias at emergence (which is not excluding the previous arguments) is higher costs for producing a male. These costs may accrue due to longer developmental phases, or differential size. None of this is known for bat flies at this point. The observed secondary female bias in our data stands in contrast to two other (albeit much smaller) samples from Fritz [23], who found an equal sex ratio at emergence in *Trichobius joblingi* Wenzel, and *Speiseria ambigua* Kessel from bats in Costa Rica. This may point to a greater flexibility in sex ratios among populations and species than previously expected.

Reproductive competition among siblings or relatives can also be a source for biased sex ratios [35]. To produce a female bias, one would have to invoke a local resource competition between males, but none between females, as sometimes observed when mating occurs at least partially before dispersal. In bat flies, mating occurs after dispersal to a host, for both sexes. Thus, both females and males are randomized among bats, parents and offspring are likely not clustered, and direct sibling competition for mates and resources is projected to be negligible [3], [36], [37].

### Operational Sex Ratio (OSR)

**Figure 6:**
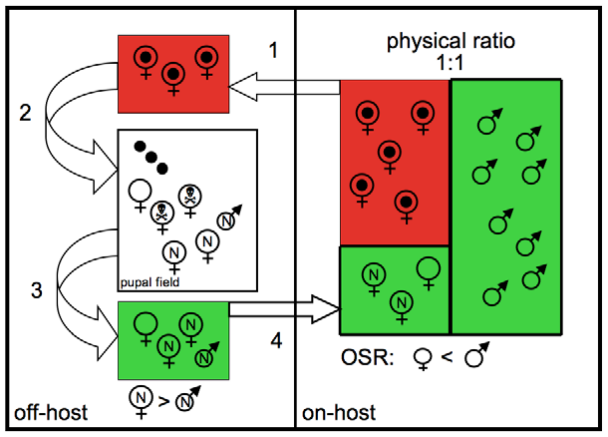
Schematic diagram comparing parasite demography on-host (right panel), and off-host (left panel). **On-host** a physical ratio of 1∶1 is observed. Red rectangle represents females with developing offspring, which are not available for reproduction. Females within green rectangle are a mixture of returning females, and teneral females (N). The green rectangles (male and female) on-host represent the operational sex ratio (OSR), which is likely male biased. **Off-host**, depositing females leave the hosts (1), and arrive at the pupal field (2), where they give birth to their offspring. Some die due to predation (skull), while new females and males emerge leave the field (3) and return to the general host population (4). Mating ensues, and females become unavailable for mating. The ratio of males and females at emergence is skewed towards females.

Previous studies asserted that males prefer virgin females (tenerals) as mates [8]. Because the bulk of mating activity is reported to occur on roosting bats, it is likely that the operational sex ratio is in fact male biased (Table 1, Figure 6). The rationale for this assertion lies in the fact that although the physical sex ratio on roosting bats is at equilibrium, females with developing offspring will be unavailable for mating, because each new offspring requires a separate insemination event, and males are not attracted to “pregnant” females [8]. Thus it is likely, that all males compete for a subset of available females, which is mainly composed of teneral females (Figure 6). This scenario also predicts that males are the predominant competitors in this system, have a lower parental investment, as well as a higher potential to mate [4]. Given that reproductive behavior seems to be very similar across bat fly species, a male biased OSR may be a more general trend in bat flies than previously assumed.

### General trends for parasites

#### Spatial and temporal stratification of sex ratios

The classical view of parasite sex ratios understands and explains their dynamics primarily in the context of the host. This may be largely appropriate for permanent parasites, when all developmental stages are host associated (e.g., lice). However, a majority of eukaryotic parasites living on or in invertebrate and vertebrates have free-living (off-host) developmental stages. Because bat flies spent a substantial amount of their life off their hosts, they exemplify such parasites. Our results point to a strong spatial and temporal heterogeneity in their daily and developmental ecologies, due to continued interactions with the larger (non-host) environment. This heterogeneity will influence male and female distribution of any such parasite within the landscape of their ecosystem. Results from this study support this assertion. When comparing temporal patterns of bat fly sex differentials on bats, it becomes apparent that foraging bats (night) exhibit a male skew, while parasite sex distribution on roosting bats (day) is near equality (Table 1, Figure 6). Similarly, when comparing spatial patterns of physical sex ratios assessed at the same time (foraging) in the global parasite environment, one finds a male skew on bats, but a female skew on the pupal field (Figure 6). If not taken into account, it is possible that demographic sampling biases are created and perpetuated. For practical applications, this also means a likely underestimation of average parasite load in a host population.

Under such circumstances, physical parasite sex ratios assessed only from hosts (as is the case in most parasitological studies) are likely to not represent a valid proxy for operational sex ratios [38]–[40]. Operational sex ratios however, are key factors in understanding the intensity and direction of sexual selection in a population. Therefore, we would like to point out that it is important to explore parasite reproduction and sex ratio in the context of host and non-host environments.

### Predation and developmental sex ratios

In our previous argument we outline that parasite systems with off-host development likely have a spatial and temporal stratification of their population. This is predicted to be particularly strong in cases where off-host ecologies do not overlap with host ecologies, as is the case for bat flies (Figure 3A), as well as many other parasites. In such cases, off-host development may necessitate a repeated period of adult relocation (mostly reproductively active females) to the greater environment, and/or other hosts. This will increase the risk of mortality for the migrating party. Furthermore, off-host developmental stages may be exposed to a predation risk similar to non-parasitic systems. In any case, it is frequently overlooked that off-host predation will play a major role in shaping physical sex ratios across developmental gradients of parasites (but see [5]).

In our study, off-host stages are exemplified by the pupae. Pupal mortality is reported at 15.5%, which is higher than the previously estimated 12% by Overal [21]. Still, our results are likely an underestimation, because we could not record mortality on the pupal field directly. Our observations of both predatory mites, and fungi as a cause of pupal mortality echo previous anecdotal observations by Fritz [23] in Costa Rica (Figure 2, A–D).

Since males mostly stay with the hosts throughout their lifetime, their risk of mortality computes primarily from their initial host seeking event as a teneral fly, and regular mortality due to age, or possibly grooming. Comparatively, the probability of death is drastically higher for females, and should positively correlate to the distance of movement from the host. Female risk is multiplicative over their life span, and specifically relates to the chance of death on the way to the pupal deposition site, at the pupal deposition site, and on the way back to the host. Thus, similar to other publications, we also predict that average male longevity in the population is higher than that of females, but without invoking selective grooming of larger females by the host bats, as suggested by Dick and Patterson [7]. Grooming is known to control the population size of ectoparasites [41]–[43], and it has been shown that larger lice are more likely to be groomed off [44]. Grooming activities cannot easily be assessed in a natural system, and empirical evidence to support sex-dependent grooming impact on bat flies is missing. Furthermore, the previously proposed grooming theory rests entirely on sexual size dimorphism. However, there are also size differences across species [7]. Therefore, both sexes of one species may be smaller than those of another species. If these two species co-occur (e.g. *Nycterophilia* sp. and *Trichobius* sp., Material and Methods), it would then lead to grooming of both sexes of the bigger species over the smaller species, and ultimately lead to size-dependent parasite control across species, rather than a biased sex ratio within a species.

Based on our natural-history observations, it is also likely that the mortality of depositing females is higher than that of teneral flies of both sexes. Our rationale is that previously fed adult bat flies (regardless of sex) have a low tolerance for starvation. Therefore there is a strong need for host relocation [8], [23]. Teneral flies on the other hand may survive up to two days without a blood meal [8]. Due to the diurnal rhythms of reproduction, emergence of tenerals peaks at a later time from pupal deposition (Figure 1C, Figure 5). Thus, predators (such as *Tetramorium* ants) may have already fed on depositing females, and collected recently deposited pupae, possibly increasing survival of tenerals. Moreover, we predict that because the ratio at emergence is female biased, there is a greater likelihood that a dead female is replaced by a teneral female. If the female bias at emergence cancels female mortality on the population level, the system may approach demographic stability (Figure 6).

Both, pupal and female adult mortality takes place after parental investment, and therefore will not influence the ESS sex allocation. In other words, while the likelihood of any female reproducing decreases, the reproductive success of surviving females increases. These values should cancel each other out, and no influence should be exerted on the average fitness of a female [2], [45].
